# Supplementary material for: Emergence of highly virulent Aeromonas dhakensis in channel catfish aquaculture: Genomic insights into pathogenicity and antimicrobial resistance
Source: Virulence. 2025 Jul 2;16(1):2525933. doi: 10.1080/21505594.2025.2525933 (PMC12233899; doi:10.1080/21505594.2025.2525933)
Supplement: Manuscript V3_Marker.docx [file KVIR_A_2525933_SM5140.docx]

**Emergence of highly virulent *Aeromonas dhakensis* in channel catfish aquaculture: genomic insights into pathogenicity and antimicrobial resistance**

Yuanyuan Wang ^1, †^, Zhengqi Feng ^1, †^, Wenbo Wu ^1^, Zhipeng Zhan ^1^, Jiaming Huang ^2^, Changjun Guo ^1,^ *, Jianguo He ^1^

^1^ School of Marine Sciences, State Key Laboratory for Biocontrol & Southern Maine Science and Engineering Guangdong Laboratory (Zhuhai), Guangdong Province Key Laboratory of Aquatic Economic Animals / Guangdong Provincial Observation and Research Station for Marine Ranching of the Lingdingyang Bay, Sun Yat-sen University, 135 Xingang Road West, Guangzhou 510275, PR China.

^2^ Guangdong Evergreen Feed Industrial Co., Ltd., Zhanjiang 524002, China.

^†^ These authors contributed equally to this work.

^＊^Corresponding author: Changjun Guo. Email: [gchangj@mail.sysu.edu.cn](mailto:gchangj@mail.sysu.edu.cn). ORCID: 0000-0003-3223-6176.

**ABSTRACT**

*Aeromonas dhakensis* has emerged as a significant pathogen in aquaculture, causing severe disease outbreaks and resulting in substantial economic losses. However, its pathogenic mechanism and virulence factors remain largely unexplored. In this study, we isolated a highly virulent strain of *A. dhakensis*, CWH5, from a severe disease outbreak in farmed channel catfish (*Ictalurus punctatus*). Through comprehensive whole-genome analysis, we elucidated its pathogenicity and the genetic basis for its high virulence and multi-antimicrobial resistance in channel catfish. Experimental infections showed that CWH5 exhibited exceptional virulence, with an LD_50_ of (5.37±0.31) ×10^5^ CFU/fish and causing 100% mortality within 24 h at a concentration of 10⁷ CFU/fish. Histopathological examinations revealed severe multi-organ damage, including extensive hepatocellular necrosis, gill epithelial destruction, and fin tissue deterioration. Whole-genome sequencing revealed a 4.92 Mb circular chromosome encoding sophisticated virulence mechanisms, such as complete Type III, IV, and VI secretion systems, and a vast arsenal of 60 antibiotic resistance genes across 15 drug classes. Comparative genomic analysis positioned CWH5 within the *A. dhakensis* clade, sharing the highest sequence similarity with *A. dhakensis* CIP 107500ᵀ. The co-localization of virulence and resistance determinants within mobile genetic elements suggests the potential for horizontal gene transfer. Our work underscored the importance of *A. dhakensis* CWH5 as an emerging pathogen in channel catfish aquaculture, providing crucial insights into the molecular mechanisms of its exceptional virulence and implying significant implications for disease management and antimicrobial resistance surveillance in aquaculture settings.

**Keywords:** *Aeromonas dhakensis*, channel catfish, aquaculture, virulence factors, antibiotic resistance, pathogenicity.

**Introduction**

Aquaculture plays a pivotal role in meeting the protein demands for the expanding global population. Among various species, the channel catfish (*Ictalurus punctatus*) being an important global aquaculture, particularly in the United States and China [1]. However, the intensification of aquaculture practices has resulted in a surge of disease outbreaks, particularly bacterial infections, threatening the sustainability of the industry [2]. Of particular concern are bacteria belonging to the genus *Aeromonas*, which are widespread in aquatic environments and can cause severe infections in various fish species [3].

Since its identification in 2002, *Aeromonas dhakensis*, initially misclassified as *A. hydrophila*, has emerged as a distinct and highly virulent species with unique genomic and phenotypic characteristics [4]. Recent studies have demonstrated its emergence as a highly virulent pathogen affecting both aquatic animals and humans [5]. While *A. dhakensis* infections have been documented in various fish species, including tilapia and carp [6, 7], its impact on channel catfish aquaculture remains poorly understood despite the economic importance of this fish species.

The pathogenicity of *Aeromonas* species involves complex mechanisms, including various virulence factors such as adhesins, toxins, secretion systems, and enzymes [8]. Recent studies have highlighted the particular importance of type III and type VI secretion systems in *A. dhakensis* pathogenicity [9]. Additionally, biofilm formation and quorum-sensing molecules have been implicated in enhancing virulence and environmental persistence of *Aeromonas* species [10].

A growing concern in aquaculture is the rising incidence of antibiotic resistance among *Aeromonas* species [11]. The extensive use of antimicrobials in aquaculture has contributed to the selection and spread of multidrug-resistant strains, limiting treatment options and potentially facilitating resistance gene transfer to other pathogens [12]. Studies have documented an increase in multidrug-resistant *Aeromonas* strains in aquaculture settings, with multiple isolates showing resistance to three or more antibiotic classes [13, 14]. Investigations have shown that *A. dhakensis* possesses various virulence mechanisms, including type III and type VI secretion systems, which contribute to host-pathogen interactions [15]. These findings suggest that *A. dhakensis* may employ unique virulence mechanisms that contribute to its success as a pathogen.

Despite these advances in understanding *A. dhakensis* pathogenicity, significant knowledge gaps remain regarding its impact on channel catfish aquaculture, specifically: strain-specific virulence mechanisms and their role in disease progression, comparative efficacy of current treatment protocols between *A. dhakensis* and *A. hydrophila* infections, and epidemiological patterns of transmission in intensive aquaculture settings. In this study, we isolated and characterized a highly virulent strain CWH5 from a farmed channel catfish. Our study aimed to investigate the clinical manifestations (including severe skin ulceration, fin erosion, and systemic hemorrhage) and histopathological changes (characterized by extensive hepatocellular necrosis, gill epithelial destruction, and inflammatory cell infiltration), assess virulence potential through *in vivo* challenges, analyze the genome for virulence factors and resistance genes, and determine antibiotic susceptibility. This comprehensive analysis provides critical insights into the pathogenic mechanisms of *A. dhakensis* in channel catfish, with implications for disease management in aquaculture.

**Materials and methods**

**Fish**

Channel catfish (*Ictalurus punctatus*) fingerlings (8-12 cm in length, 25±3 g) were obtained from a commercial farm in Guangdong Province, China. Fish were acclimated for two weeks in 145-L tanks with stocking density of 20 fish/m³. Water quality parameters were maintained at: temperature 25±1°C, dissolved oxygen >5 mg/L, pH 7.2±0.2, ammonia-N <0.02 mg/L, and nitrite-N <0.01 mg/L. Water was partially (30%) exchanged daily. Fish were fed commercial pellets (32% protein) at 2% body weight twice daily.

***Bacterial isolation and identification***

In June 2024, tissue samples were collected from moribund channel catfish (*I. punctatus*) during a severe disease outbreak in Zhuhai, Guangdong Province, China. Fish showing clinical signs of disease (lethargy, skin ulcers, fin erosion, and hemorrhages) were sampled. Tissue samples were collected from both external sites (skin, fins, gills) and internal organs (liver, spleen, kidney, intestine). Samples were taken from ulcerated areas, fins, gills, liver, spleen, kidneys, and intestines, as these tissues represent key sites for bacterial invasion and dissemination during systemic infections. These organs are particularly relevant as they show characteristic pathological changes during *A. dhakensis* infections. All samples underwent surface disinfection with 70% ethanol, followed by homogenization in sterile PBS. The homogenates were streaked onto TSA plates and incubated at 28°C for 24-48 h. Dominant colonies were subsequently purified through repeated streaking.

Genomic DNA was extracted using the bacterial DNA extraction kit (Omega, USA) following the manufacturer's instructions. The 16S rRNA gene was amplified using universal primers 27F and 1492R. Sequencing was performed by RuiBiotech (Beijing, China) using the Illumina NovaSeq platform with 150 bp paired-end reads. Average sequencing depth was 100X with Q30>90%. And sequences were analyzed using BLAST against the GenBank database. Phylogenetic analysis was performed using FastME 2.1.6.1 based on GBDP (Genome BLAST Distance Phylogeny) distances calculated from 16S rDNA sequences and whole genome sequences. The branch lengths were scaled according to GBDP distance formula d5. The phylogenetic tree reliability was evaluated using 100 pseudo-bootstrap replicates, with support values >60% displayed. Trees were rooted at the midpoint following Farris's method [16].

***Phenotypic and biochemical characterization***

Metabolic characterization was performed using Biolog GEN III MicroPlate™ (Biolog Inc., USA). Bacterial suspensions were prepared in IF-B inoculating fluid. The optical density was measured and adjusted to 95% transmittance using a SYNERGY NEO2 microplate reader (BioTek Instruments, USA) at OD600. The standardized bacterial suspensions were then incubated at 28°C for 24 h. The transmittance was calibrated using clean, uninoculated IF-B fluid as a blank control, with separate blanking for each inoculation tube to account for potential optical variations. The turbidity meter was regularly calibrated using standard turbidity tubes (85%T or 65%T) according to manufacturer specifications. This standardized procedure ensured consistent bacterial suspension preparations across all experiments. Results were analyzed using the Biolog Microbial Identification System software [17].

Antibiotic susceptibility was determined using the Kirby-Bauer disc diffusion method [18] on Mueller-Hinton agar (Oxoid, UK) according to the Clinical and Laboratory Standards Institute guidelines (CLSI, 2020). Antibiotic susceptibility testing was performed against 38 antibiotics categorized (Hangzhou Binhe Microbiology Co., Ltd., China) by their regulatory approval status. These included three aquaculture-approved antibiotics (florfenicol, doxycycline, and enrofloxacin) as per the 2024 Chinese Aquaculture Medicine White Paper, two prohibited agents (chloramphenicol and furazolidone), and 33 clinically relevant antibiotics. The clinical antibiotics comprised β-lactams (*n*=12), aminoglycosides (*n*=5), tetracyclines (*n*=2), macrolides (*n*=2), quinolones (*n*=5), and others (*n*=7). This comprehensive panel was strategically designed to evaluate current therapeutic options, regulatory compliance, and potential antimicrobial resistance patterns, particularly considering the zoonotic implications of *A. dhakensis.* The plates were incubated at 28°C for 24 h, and inhibition zones were measured. The results were interpreted as susceptible (S), intermediate (I), or resistant (R). *Escherichia coli* ATCC 25922 (Zuoke Biological Technology, China) served as the quality control strain.

***Experimental infections***

Healthy channel catfish (*Ictalurus punctatus*, 8-12 cm in length, 25±3 g in weight, mixed sex) were obtained from a farm in Guangdong province, China. Fish were acclimated for two weeks in 145-L tanks with aerated, dechlorinated water at 25±1°C, pH 7.2±0.2, and dissolved oxygen >5 mg/L. They were fed commercial pellets at 2% of their body weight daily. Feeding was suspended 24 h prior to bacterial challenge and resumed 24 h post-infection, with the same feeding rate (2% body weight daily) maintained throughout the 10-day observation period. This standardized feeding protocol was used for all treatment groups to keep normal physiological conditions and avoid stress from lack of food. Throughout the experiment, water quality parameters were monitored daily and maintained at optimal levels (temperature: 25±1°C, dissolved oxygen: >5 mg/L, pH: 7.2±0.2). All animal experiments were conducted following Sun Yat-sen University's guidelines (No. 2024061601).

Bacterial suspensions were prepared in sterile PBS at concentrations of 1×10⁷, 1×10⁶, 1×10⁵, 1×10⁴ and 1×10^3^ CFU/fish following ISO 7218:2007 standards. 35 fish in each of five groups were randomly assigned to receive an intraperitoneal injection of 0.1 mL of bacterial suspension or sterile PBS (control), based on previous similar pathogenicity studies. Mortality was monitored for 10 days post-injection. The survival assays were performed in triplicate with 35 fish per group in each independent experiment. Mortality rates are presented as mean ± standard deviation (SD). The half-maximal lethal dose (LD_50_) was measured by the Bliss method. Challenge experiments were conducted in triplicate with 35 fish per treatment group in each replicate.

***Histopathology***

For histopathological analysis, tissue samples were collected from moribund fish (*n*=3) at 24 h post-infection from the group challenged with 1×10⁶ CFU/fish of *A. dhakensis* CWH5. Matching tissue samples were also collected from PBS-injected control fish (*n*=3) at the same time points. Three independent biological replicates were performed. Tissue samples from the liver, gills and fins of diseased fish were fixed in 10% neutral buffered formalin for 24 h. The fixed samples were processed, embedded in paraffin, sectioned at 5 μm thickness, and stained with hematoxylin and eosin (H&E). The sections were examined using the Aperio VERSA 8 FISH digital pathology scanner (Leica, Germany).

***Genomic analyses***

Genomic DNA of isolate CWH5 was extracted using the Omega Bacterial DNA Kit (Omega, USA) according to the manufacturer's instructions. DNA quality was assessed using a NanoDrop 2000 spectrophotometer (Thermo Fisher Scientific, USA) and Qubit 4.0 Fluorometer (Invitrogen, USA). Whole-genome sequencing was performed using the PacBio Sequel II platform (Pairsonne Biotechnology Co., Ltd, Shanghai, China). *De novo* assembly was performed using Unicycler v0.4.8 and Flye v2.9.1 [19, 20], with base correction using Pilon software [21]. Gene prediction was carried out using Glimmer 3.02 [22], while rRNA and tRNA genes were identified using RNAmmer [23]and tRNAscan-SE [24], espectively. Genome annotation was performed using the NCBI PGAP [25], and the genome was visualized using CGView [26]. Average nucleotide identity (ANI) values were calculated using the JSpeciesWS [27]. Tree inferred with FastME 2.1.6.1 [16] from GBDP distances calculated from genome sequences. The branch lengths are scaled in terms of GBDP distance formula d5. The numbers above branches are GBDP pseudo-bootstrap support values > 60% from 100 replications, with an average branch support of 95.6%. The tree was rooted at the midpoint. Scale bar represents 0.010 substitutions per nucleotide position.

Functional annotation was performed using the following databases: Kyoto Encyclopedia of Genes and Genomes (KEGG) [28]. Non-Redundant Protein Database (NR), Clusters of Orthologous Groups (COG) [29] and Carbohydrate-Active Enzymes Database (CAZy) [30]. Pathogenicity-related genes were identified using the Pathogen-Host Interaction database (PHI-base) [31]. Virulence factors were predicted using the Pathogenic Bacteria Virulence Factor Database (VFDB) [32], which contains 74 bacterial genes, 532 experimentally verified virulence factors, and 2,599 virulence factor-related genes. BLAST analysis was performed using stringent criteria: E-value ≤1e-5, amino acid sequence identity ≥60%, alignment coverage ≥70%, and gap length <10% of total alignment. Antibiotic resistance genes were identified using the Comprehensive Antibiotic Resistance Database (CARD) [33] with parameters: E-value ≤1e-6, amino acid sequence identity ≥45%, and alignment coverage of ≥70%.

***Statistical Analysis***

All experiments were performed in triplicate unless otherwise stated. Survival data were analyzed using Kaplan-Meier method with log-rank test. The LD_50_ values were calculated using the Bliss method and expressed as mean ± standard deviation (SD) with 95% confidence intervals (CI). Mortality rates from the bacterial challenge experiments are presented as mean ± SD based on three independent experiments with 35 fish per group. Histopathological analyses were performed on tissue samples from three biological replicates per group, with representative images shown. Semi-quantitative analysis of tissue damage was not performed, as the study focused on qualitative description of histopathological changes. Statistical analyses were performed using GraphPad Prism 9.0 (GraphPad Software, USA).

**Results**

***Identification and characterization of a highly virulent A. dhakensis CWH5***

During a severe disease outbreak characterized by high mortality in farmed channel catfish, we isolated and identified an aggressive strain of *A. dhakensis*, designated CWH5. Clinical examination revealed severe pathological manifestations, including extensive skin erosion, pronounced fin congestion, and maxillary ulceration (Figure 1A), indicating the development of systemic infection in affected fish. Molecular and phylogenetic analyses based on 16S rDNA positioned strain CWH5 within a well-supported clade of *A. dhakensis* (bootstrap values >70%), where it clustered closely with known virulent strains (Figure 1B). The metabolic profiling using Biolog GEN III revealed strong utilization of tissue-associated carbohydrates (dextrin, D-maltose, D-trehalose) and amino acids (Figure 1C, Table S1), suggesting metabolic adaptations that could facilitate host colonization and persistence.

***Experimental infection reveals exceptional virulence***

Systematic infection studies were conducted to quantitatively assess the pathogenic potential of CWH5 in channel catfish. The results revealed striking virulence characteristics, with a remarkably low LD_50_ of 5.37 ± 0.31 × 10^5^ CFU/fish (95% CI: 4.78-5.96 × 10^5^) (Table 1). Survival analysis demonstrated a clear dose-dependent mortality pattern (Figure 1D). The highest concentration (1×10⁷ CFU/fish) resulted in 100% mortality within 24 h post-infection, while moderate doses (1×10⁶ CFU/fish) maintained considerable virulence with 65.71 ± 2.86% mortality. Lower concentrations showed reduced effects, with 1×10⁵ CFU/fish causing 18.57 ± 1.43% mortality, and minimal doses (1×10⁴ and 1×10³ CFU/fish) inducing no mortality. Control groups administered PBS remained unaffected throughout the observation period.

**Table 1** Determination of median lethal dose (LD_50_).

| **Bacteria strain** | **Infection dose (CFU/fish)** | **Mortality rate (%)** | **LD_50_**  **CFU/fish** |
| --- | --- | --- | --- |
| CWH5 | 1 × 10⁷ | 100 ± 0.00 | (5.37 ± 0.31) ×10^5^  (95% CI:  4.78-5.96 × 10^5^) |
|  | 1 × 10^6^ | 65.71 ± 2.86 |  |
|  | 1 × 10^5^ | 18.57 ± 1.43 |  |
|  | 1 × 10^4^ | 0 ± 0.00 |  |
|  | 1 × 10^3^ | 0 ± 0.00 |  |
| PBS | - | 0 ± 0.00 | - |

***Histopathological evidence of aggressive tissue invasion***

Histopathological examination revealed distinct differences between control and infected fish tissues. Control fish maintained normal tissue architecture, characterized by well-organized hepatocyte cords and clear sinusoidal spaces in liver (Figure 2C), distinct primary filaments and secondary lamellae with regular epithelial cells in gills (Figure 2E), and organized epithelial layers with intact connective tissue in fins (Figure 2G). Infected fish tissues showed severe pathological changes across multiple organs. The liver exhibited extensive hepatocellular necrosis with pyknotic nuclei, cell swelling, and pronounced inflammatory infiltration (Figure 2D). Gill tissues displayed significant epithelial damage and lamellar fusion (Figure 2F), potentially compromising respiratory function. Fin sections showed severe structural disruption, characterized by extensive tissue loss and inflammatory cell infiltration (Figure 2H). These pathological changes indicate CWH5's aggressive tissue invasion capabilities and the strong host inflammatory response to infection.

The widespread and severe tissue damage observed across multiple organs indicates CWH5's aggressive invasion capabilities and diverse virulence mechanisms. These histopathological findings correlate with the gross pathological observations, including skin lesions, fin congestion, and intestinal inflammation (Figure 2A, B), underscoring the strain's potential as a highly virulent pathogen in channel catfish aquaculture.

***Genomic analysis reveals distinctive features of A. dhakensis CWH5***

Genomic analysis of CWH5 revealed a single circular chromosome (4,916,354 bp, G+C content 61.65%) containing 4,346 predicted ORFs (Table 2, Figure 3A). The genome includes multiple RNA elements (10 copies each of 16S and 23S rRNA genes, 11 copies of 5S rRNA, and 126 tRNA genes) and regulatory features (55 ncRNA genes, 5 CRISPR arrays, and 353 repeat regions), indicating sophisticated gene regulation and defense mechanisms.

**Table 2** Genome structural characteristics of *Aeromonas dhakensis* CWH5.

| **Property** | **Value** |
| --- | --- |
| Genome Length | 4,916,354 bp |
| Number of scaffold(contig) | 1 |
| G+C content | 61.65% |
| Num of ORF | 4346 |
| ncRNA | 55 |
| 5S rRNA | 11 |
| 16S rRNA | 10 |
| 23S rRNA | 10 |
| tRNA | 126 |
| CRISPRs | 5 |
| Repeats | 353 |

Comparative genomic analyses positioned CWH5 within the Aeromonas genus, with ANI analysis showing highest sequence similarity to A. dhakensis CIP 107500ᵀ (97.25%, aligned 88.47%) and A. dhakensis SSU (97.12%, aligned 88.85%) (Figure 3B, Table S2). Digital DNA-DNA hybridization (dDDH) analysis confirmed these findings, with values of 76.6% for *A. dhakensis* CIP 107500 and 76.1% for *A. aquariorum* CECT 7289, while other Aeromonas species showed significantly lower values (30.1-50.6%) (Table 3). Core genome phylogenetic analysis generated a well-supported maximum-likelihood tree (bootstrap >95%), placing CWH5 in a distinct clade with other *A. dhakensis* strains (Figure 3C). The strain formed a monophyletic group with *A. dhakensis* CIP 107500 and *A. aquariorum* CECT 7289, with clear phylogenetic separation from other *Aeromonas* species.

**Table 3** Calculated dDDH values between *Aeromonas dhakensis* CWH5 and homologous bacteria.

| **Query strain** | **Subject strain** | **dDDH (d4, in %)** | **C.I. (d4, in %)** |
| --- | --- | --- | --- |
| *Aeromonas dhakensis* CWH5 | *A. dhakensis* CIP 107500 | 76.6 | [73.6 - 79.3] |
|  | *A. aquariorum* CECT 7289 | 76.1 | [73.1 - 78.9] |
|  | *A. hydrophila* ATCC 7966 | 50.6 | [47.9 - 53.2] |
|  | *A. hydrophila* subsp. ranae CIP 107985 | 50.3 | [47.7 - 53.0] |
|  | *A. bestiarum* CECT 4227 | 34.2 | [31.8 - 36.7] |
|  | *A. piscicola* LMG 24783 | 33.4 | [31.0 - 35.9] |
|  | *A. taiwanensis* LMG 24683 | 32.8 | [30.4 - 35.3] |
|  | *A. hydrophila* subsp. anaerogenes CECT 4221 | 32.6 | [30.2 - 35.1] |
|  | *A. caviae* NCTC 12244 | 32.6 | [30.2 - 35.1] |
|  | *A. sanarellii* LMG 24682 | 32.5 | [30.1 - 35.0] |
|  | *A. media* CECT 4232 | 31.2 | [28.8 - 33.7] |
|  | *A. enteropelogenes* CECT 4487 | 31.1 | [28.7 - 33.7] |
|  | *A. trota* CECT 4255 | 31 | [28.6 - 33.5] |
|  | *A. jandaei* CECT 4228 | 30.7 | [28.3 - 33.2] |
|  | *A. encheleia* CECT 4342 | 30.3 | [27.9 - 32.8] |
|  | *A. veronii* CECT 4257 | 30.1 | [27.7 - 32.6] |

***Metabolic and functional genomic attributes underlying virulence***

Functional genomic analysis revealed CWH5's comprehensive metabolic capabilities. GO analysis demonstrated significant enrichment in cellular nitrogen compound metabolism (3,079 genes), biosynthetic processes (2,072 genes), membrane-associated proteins (1,188 genes), and DNA binding functions (2,034 genes) (Figure 4A). KEGG pathway analysis identified complete systems for central carbon metabolism, membrane transport, and regulatory processes, with notable enrichment in signaling and cellular processes (Figure 4B).

The strain possesses an extensive repertoire of carbohydrate-active enzymes, particularly Glycoside Hydrolases (GH) and Glycosyl Transferases (GT) (Figure 4C). COG functional classification revealed balanced distribution across major cellular functions, with significant representation in amino acid transport, transcription, and energy production (Figure 4D). PHI database analysis identified 684 genes associated with hypervirulence and 312 genes classified as unaffected in pathogenicity, along with numerous chemical sensitivity/resistance determinants (Figure 4E), suggesting sophisticated host interaction capabilities.

***Antibiotic resistance profile and associated genetic determinants***

*A. dhakensis* CWH5 exhibited extensive antimicrobial resistance against multiple clinically important antibiotics (Table 4). Phenotypic testing revealed complete resistance to penicillins (penicillin, oxacillin, ampicillin, amoxicillin), correlating with multiple *β*-lactam resistance genes including *AQU-1*, *OXA-12*, and *cphA7*. The strain showed selective resistance to cephalosporins, remaining resistant to early-generation agents while maintaining susceptibility to later-generation compounds. Notable resistance was observed against protein synthesis inhibitors (erythromycin, azithromycin, chloramphenicol, clindamycin), corresponding to 12 macrolide resistance genes and 11 phenicol resistance determinants.

**Table 4.** Antibiotic susceptibility profile of *Aeromonas dhakensis* CWH5 determined by disc diffusion assay.

| **Antibiotics** | **Inhibition zone diameter/mm** | **Sensitivity** | **Antibiotics** | **Inhibition zone diameter/mm** | **Sensitivity** |
| --- | --- | --- | --- | --- | --- |
| penicillin | 0 | R | ciprofloxacin | 33 | S |
| oxacillin | 0 | R | lincomycin | 0 | R |
| ampicillin | 0 | R | vancomycin | 10 | R |
| piperacillin | 25 | I | polymyxin b | 17 | I |
| cephalexin | 0 | R | compound sulfamethoxazole | 0 | R |
| cefazolin | 0 | R | chloramphenicol | 14 | R |
| cefuroxime | 27 | S | clindamycin | 0 | R |
| ceftazidime | 30 | S | levofloxacin | 33 | S |
| ceftriaxone | 27 | S | imipenem | 14 | R |
| cefoperazone | 25 | I | doxycycline | 12 | I |
| amikacin | 25 | I | florfenicol | 10 | R |
| gentamicin | 24 | S | cefotaxime | 31 | S |
| kanamycin | 26 | S | lomefloxacin | 32 | S |
| streptomycin | 16 | S | ofloxacin | 32 | S |
| tetracycline | 11 | R | neomycin | 23 | S |
| minocycline | 22 | S | enrofloxacin | 32 | S |
| erythromycin | 0 | R | amoxicillin | 0 | R |
| azithromycin | 10 | R | aztreonam | 42 | S |
| norfloxacin | 31 | S | furazolidone | 23 | S |

**Table 5** Distribution statistics of cards and vfs on gene islands.

| **Molecule** | **Gene** | **Start** | **End** | **Strand** | **Description** |
| --- | --- | --- | --- | --- | --- |
| chr:Is2(1509432-1525507) | vfdb | 1509432 | 1510295 | forward | glucose-1-phosphate thymidylyltransferase RfbA |
| chr:Is2(1509432-1525507) | vfdb | 1510307 | 1510858 | forward | dTDP-4-dehydrorhamnose 3,5-epimerase |
| chr:Is2(1509432-1525507) | vfdb | 1519086 | 1520480 | forward | mannose-1-phosphate guanylyltransferase |
| chr:Is6(1976093-1981754) | vfdb | 1976093 | 1977412 | forward | type VI secretion system polymorphic nuclease effector |
| chr:Is6(1976093-1981754) | vfdb | 1977608 | 1978411 | forward | PoNi-like cognate immunity protein |
| chr:Is6(1976093-1981754) | vfdb | 1978697 | 1979080 | forward | hypothetical protein KBAD45_24040 |
| chr:Is6(1976093-1981754) | vfdb | 1979241 | 1980593 | forward | tetratricopeptide repeat protein |
| chr:Is6(1976093-1981754) | vfdb | 1981353 | 1981754 | forward | type VI secretion protein |
| chr:Is10(2962621-2973709) | vfdb | 2963629 | 2964804 | forward | UDP-N-acetylglucosamine 2-epimerase (non-hydrolyzing) |
| chr:Is10(2962621-2973709) | vfdb | 2964872 | 2966122 | forward | UDP-N-acetyl-D-mannosamine dehydrogenase |
| chr:Is15(3355032-3391595) | card | 3383530 | 3383937 | forward | H-NS family nucleoid-associated regulatory protein |
| chr:Is16(3669076-3677135) | vfdb | 3677132 | 3679879 | reverse | type VI secretion system tip protein VgrG |
| chr:Is19(4435949-4449552) | card | 4443997 | 4445955 | reverse | elongation factor G |
| chr:Is21(4586553-4601552) | vfdb | 4585552 | 4586556 | forward | UDP-N-acetylglucosamine 4,6-dehydratase |
| chr:Is23(4874698-4909483) | card | 4884490 | 4885704 | forward | chloramphenicol/florfenicol efflux MFS transporter FloR |
| chr:Is23(4874698-4909483) | card | 4886304 | 4887578 | reverse | tetracycline repressor protein TetA |
| chr:Is23(4874698-4909483) | card | 4887609 | 4888259 | forward | tetracycline resistance transcriptional repressor TetR(A) |
| chr:Is23(4874698-4909483) | card | 4891524 | 4892021 | forward | trimethoprim-resistant dihydrofolate reductase DfrA12 |
| chr:Is23(4874698-4909483) | card | 4892441 | 4893220 | forward | Streptomycin 3''-adenylyltransferase, partial |
| chr:Is23(4874698-4909483) | card | 4893725 | 4894564 | forward | sulfonamide-resistant dihydropteroate synthase Sul1 |
| chr:Is23(4874698-4909483) | card | 4899875 | 4900780 | reverse | Mph(A) family macrolide 2'-phosphotransferase |
| chr:Is24(4879360-4902217) | card | 4884490 | 4885704 | forward | chloramphenicol/florfenicol efflux MFS transporter FloR |
| chr:Is24(4879360-4902217) | card | 4886304 | 4887578 | reverse | tetracycline repressor protein TetA |
| chr:Is24(4879360-4902217) | card | 4887609 | 4888259 | forward | tetracycline resistance transcriptional repressor TetR(A) |
| chr:Is24(4879360-4902217) | card | 4891524 | 4892021 | forward | trimethoprim-resistant dihydrofolate reductase DfrA12 |
| chr:Is24(4879360-4902217) | card | 4892441 | 4893220 | forward | Streptomycin 3''-adenylyltransferase, partial |
| chr:Is24(4879360-4902217) | card | 4893725 | 4894564 | forward | sulfonamide-resistant dihydropteroate synthase Sul1 |
| chr:Is24(4879360-4902217) | card | 4899875 | 4900780 | reverse | Mph(A) family macrolide 2'-phosphotransferase |

Genomic analysis identified 60 resistance genes across 15 drug classes, with significant clustering in the Is23/Is24 resistance island (4874698-4909483) (Figure 5E, Table 5). The resistance arsenal includes 18 fluoroquinolone resistance genes, 16 genes each for beta-lactam and tetracycline resistance, and various other determinants (Table S3). The resistance genes comprise efflux pumps (28 genes), target alteration genes (17 genes), and antibiotic inactivation genes (8 genes), including the AcrAB-TolC efflux complex (Table S4). Phenotypic testing showed susceptibility to fluoroquinolones and aminoglycosides, suggesting complex regulatory control over resistance expression.

***Virulence determinants and pathogenicity islands***
Genomic analysis revealed CWH5's extensive repertoire of virulence factors and associated genomic islands. VFDB analysis identified motility-related genes as the predominant category (>400), particularly flagella-related components and polar flagella systems (Figure 5C and D). The strain possesses multiple key virulence genes, including T6SS spike proteins (*vgrG*1-3) showing high sequence identity (90.1-96.1%) with known virulence-associated *VgrG*s, MARTX toxin (*rtxA*, 95.2%), and various hemolysins (*ahh*1, *hly*A, *AHA_RS17655*) with >98% identity to reference sequences (Table 6).

**Table 6** Key virulence genes of *Aeromonas dhakensis* CWH5.

| **Gene** | **Function** | **Virulence** | **Identity (%)** | **Number of Copies** |
| --- | --- | --- | --- | --- |
| *rtxA* | MARTX toxin | Cytotoxicity, host cell disruption | 95.2 | 1 |
| *ahh1* | Extracellular hemolysin | Hemolysis, tissue damage | 98.5 | 1 |
| *hlyA* | Hemolysin A | Host cell lysis | 98.8 | 1 |
| *AHA_RS17655* | Hemolysin III | Pore formation, osmotic lysis | 98.5 | 1 |
| *vgrG1-3* | T6SS spike proteins | Effector delivery, antibacterial activity | 90.1 - 96.1 | 3 |
| *lfiI* | Lateral flagella | Host colonization, biofilm formation | 92.2 | 1 |

Note:"Number of Copies" column indicates the presence of multiple copies or paralogs of a given gene in the *A. dhakensis* CWH5 genome. there are three distinct *vgrG* genes (*vgrG1*, *vgrG2*, *vgrG3*) with high identity matches.

CWH5 harbors sophisticated virulence mechanisms including complete T3SS, T4SS, and T6SS, along with genes involved in lipopolysaccharide and exopolysaccharide biosynthesis, collectively enabling host interaction and immune evasion. Integrated prediction methods identified multiple virulence-associated genomic islands (Figure 5E). The Is6 region (1976093-1981754) contains a complete T6SS gene cluster with associated effector proteins, while Is2 (1509432-1525507) harbors genes for lipopolysaccharide modification, including glucose-1-phosphate thymidylyltransferase (*rfbA*) and mannose-1-phosphate guanylyltransferase, potentially enhancing immune evasion capabilities. The Is23/Is24 region (4874698-4909483) represents a multidrug resistance island housing diverse resistance determinants (*FloR*, *TetA*/*TetR*, *DfrA12*, *Sul1*).

The structural organization of these genomic islands demonstrates clustering of virulence and resistance genes (Figure 5F), frequently associated with mobile genetic elements, suggesting acquisition through horizontal gene transfer. The presence of H-NS family regulatory proteins within the Is15 island indicates sophisticated control over virulence gene expression, enabling adaptation to different host environments during infection. This genomic architecture, combining diverse virulence factors, resistance mechanisms, and regulatory systems, equips CWH5 with comprehensive pathogenic capabilities for both environmental persistence and host infection, supporting its emergence as a significant aquaculture pathogen.

**Discussion**

The exceptional virulence of *A. dhakensis* CWH5, characterized by its notably low LD₅₀ and rapid mortality kinetics, represents a significant advancement in our understanding of emerging pathogens in channel catfish aquaculture. The acute toxicity and high mortality exhibited by CWH5 align with recent pathogenicity studies of *A. dhakensis*. Recent research has demonstrated that *A. dhakensis* ST656 induces severe septicemia in striped catfish [34, 35], while an *A. dhakensis* strain from diseased *Ancherythroculter nigrocauda* exhibited an LD_50_ of 7.76×10^4^ CFU/fish [36]. Additionally, *A. dhakensis* at 2.05×10⁷ CFU/mouse caused 100% mortality within 48 hours in a mouse model [37], confirming its high pathogenicity across multiple host species.

The severe clinical manifestations and histopathological findings in CWH5 infections exceed in severity those previously reported for *A. dhakensis* [6, 7, 38, 39]. The extensive multi-organ tissue damage, particularly the pronounced hepatocellular necrosis and gill epithelial destruction, indicates aggressive tissue invasion capabilities that contrast with more localized damage typically seen in other *Aeromonas* infections [40]. Recent studies of *A. dhakensis* ST656 isolates have identified key virulence determinants including *aerA/act* (encoding aerolysin/cytotoxic enterotoxin), *ahh1* (extracellular hemolysin), and *hlyA* (hemolysin) [41]. Additionally, *A. dhakensis* uniquely possesses the *ast* gene encoding heat-stable cytotonic enterotoxin [42].

Moreover, the presence of genes encoding various toxins, including hemolysin, cytotoxic enterotoxin, and aerolysin-related toxins, in CWH5 infections aligns with the severe tissue damage and high mortality rates observed. These toxins possibly utilize various mechanisms, such as pore formation, cell lysis, and interference with host signaling pathways, to enhance the pathogen's virulence [43]. The fact that these toxin genes are found alongside components of secretion systems imply a well-orchestrated method for delivering toxin and targeting host cell. This pattern of widespread tissue invasion has important implications for disease management, as it suggests that early intervention may be crucial for treatment success and highlights the need for preventive measures such as vaccination and improved biosecurity in aquaculture settings.

The comprehensive virulence mechanisms of CWH5 are further evidenced by its sophisticated protein secretion systems. The presence of complete T2SS and T6SS in CWH5, consistent with recent finding [42], suggests advanced protein secretion mechanisms contributing to its virulence. The T6SS apparatus in CWH5 shows notable complexity, featuring three VgrG proteins (VgrG1-3) with high sequence identity (90.1-96.1%), indicates enhanced effector delivery capabilities. Recent structural studies have revealed that VgrG proteins, together with their associated chaperones, are crucial for both T6SS assembly and effector translocation [44]. Based on structural and functional studies, VgrG proteins typically form trimeric complexes acting as membrane-penetrating structures, similar to bacteriophage T4 tail spikes [45]. The crystal structure of VgrG1 from *P. aeruginosa* reveals a needle-like shape with distinct functional domains - a head domain for cargo protein scaffolding and a β-roll spike domain for membrane puncturing [46].

The strategic organization of virulence and resistance determinants within genomic islands presents unique implications for pathogen evolution and disease management. The co-localization of these traits within mobile genetic elements, a pattern observed in other emerging aquatic pathogens like *A. hydrophila* and *A. veronii* [47, 48]. suggests selective advantage for their joint inheritance and expression. The presence of H-NS family regulators within these islands indicates sophisticated virulence gene regulation [49], while multiple VgrG proteins and their associated genomic islands suggest expanded capacity for protein secretion and bacterial competition [50].

CWH5's extensive antimicrobial resistance profile, particularly against β-lactams and tetracyclines, mirrors patterns in clinical isolates [5], raising concerns for both animal and human health. The predominance of efflux-based resistance mechanisms, combined with target modification and antibiotic inactivation genes, provides robust defense against diverse drug classes. Of particular concern are resistance genes against critical aquaculture antibiotics (florfenicol-*floR*, oxytetracycline-*tetA*/*tetR*), limiting therapeutic options. Recent studies have documented similar resistance patterns in *A. dhakensis* strains from various aquaculture settings [39, 51], suggesting widespread distribution of these resistance determinants.

The metabolic versatility of CWH5 likely contributes to its pathogenic success, as evidenced by its ability to utilize diverse host-relevant substrates. The extensive array of carbohydrate-active enzymes suggests sophisticated mechanisms for nutrient acquisition and host tissue degradation [39]. These genomic adaptations, supported by Biolog GEN III phenotypic testing, indicate capabilities for both environmental persistence and efficient host colonization. Comparative genomic analysis has revealed shared virulence mechanisms with highly virulent *A. dhakensis* strains from other aquaculture species, while unique genomic islands and virulence determinants in CWH5 suggest ongoing evolution and host adaptation [51].

Based on our genomic and pathological findings, we propose a comprehensive, multi-faceted approach to control *A. dhakensis* infections in channel catfish aquaculture. This integrated strategy encompasses enhanced biosecurity measures, including all-in-all-out production systems, strict quarantine procedures for new stock, and regular PCR-based health monitoring for early detection. Disease management should focus on developing vaccines targeting conserved antigens (particularly T3SS and T6SS components), implementing strategic antibiotic rotation based on susceptibility patterns, and utilizing immunostimulants and probiotics as prophylactic measures. Environmental management requires real-time monitoring of water quality parameters, reduced stocking densities to minimize stress, and improved water circulation systems. Additionally, long-term breeding programs should be established to select for disease-resistant fish strains based on identified host response patterns, incorporating genomic selection approaches and regular evaluation of resistance traits.

However, it is important to note that the presence of virulence genes does not necessarily indicate their functional expression or contribution to pathogenicity. Further experimental validation through transcriptomic and proteomic analyses is essential to confirm the expression and role of these virulence factors during infection. Future research priorities should focus on elucidating specific host-pathogen interactions, investigating horizontal gene transfer mechanisms, developing targeted control strategies, improving early detection systems for hypervirulent strains, and understanding antimicrobial resistance evolution in aquaculture environments. The success of these measures will require consistent implementation and regular evaluation of their effectiveness. A deeper understanding of virulence and resistance evolution in aquaculture settings can inform management practices to mitigate risks. Ultimately, this integrated approach addressing pathogen evolution, host resilience, and environmental factors will be crucial for confronting the mounting challenges posed by CWH5 and related emerging pathogens.

**Conclusion**

This study presents several significant findings regarding the characterization of a novel *Aeromonas dhakensis* strain CWH5, isolated from diseased channel catfish during a severe disease outbreak. The strain demonstrated exceptional virulence, with an LD_50_ of 5.37×10⁵ CFU/fish, causing rapid and severe mortality in experimental infections. Comprehensive histopathological examination revealed extensive multi-organ damage, characterized by severe hepatocellular necrosis, gill epithelial destruction, and widespread inflammatory cell infiltration. Genomic analysis of the 4.92 Mb circular chromosome unveiled a sophisticated array of virulence determinants and antimicrobial resistance genes. Notably, we identified 60 antibiotic resistance genes spanning 15 drug classes, along with complete gene clusters encoding multiple secretion systems (T3SS, T4SS, and T6SS) and an extensive repertoire of virulence factors. Of particular significance was the discovery of resistance and virulence genes co-localized within genomic islands, suggesting potential mechanisms for the concurrent transfer of these traits. These findings provide comprehensive insights into the pathogenic potential of *A. dhakensis* CWH5 and its emergence as a significant threat to channel catfish aquaculture.

**Acknowledgements**

The authors would like to acknowledge all study participants and individuals who contributed to this study.

**Disclosure statement**

The authors declare that the research was conducted in the absence of any commercial or financial relationships that could be construed as a potential conflict of interest.

**Authorship contribution statement**

**Yuanyuan Wang**: Formal analysis; investigation, visualization; writing – original draft; writing – review and editing. **Zhengqi Feng**: Formal analysis; data curation, investigation, visualization. **Wenbo Wu**: Formal analysis; visualization. **Zhipeng Zhan**: Investigation, data curation, resources. **Jiaming Huang**: Investigation, resources. **Changjun Guo**: Conceptualization; formal analysis; funding acquisition; writing – review and editing. **Jianguo He**: Resources; funding acquisition; project administration. All authors discussed the results and commented on the manuscript.

**ORCID**

Changjun Guo, <http://orcid.org/0000-0003-3223-6176>.

**Funding**

This work was supported by the National Key Research and Development Program of China (No. 2022YFE0203900), the China Agriculture Research System (No. CARS-46), the Guangdong Key Research and Development Program (Nos. 2022B1111030001), the Guangdong Province Basic and Applied Basic Research Fund Project (No. 2023A1515110395), and the Science and Technology Plan Project of Guangdong Province (No. 2024B1212040007).

**Data Availability Statement**

The raw data that support the findings of this study have been uploaded to Figshare repository (https://doi.org/10.6084/m9.figshare.27623892.v1). The complete genomic sequences of CWH5 have been deposited in NCBI (https://www.ncbi.nlm.nih.gov/nuccore/CP170761.1/), under the accession numbers CP170761 (chromosome). Further inquiries can be directed to the corresponding author.

**Ethics statement**

The ARRIVE guidelines were followed during your study involving laboratory animals. All animal experiments were conducted in accordance with the guidelines for the Care and Use of Laboratory Animals of Sun Yat-sen University (No. 2024061601), ensuring adherence to ethical regulations for animal use. Informed consent was obtained from the owner of the fish farm in Zhuhai, Guangdong province for the use of the fish in this study.

**References**

[1] Zhong L., Song C., Chen X., et al. Channel catfish in China: Historical aspects, current status, and problems. Aquaculture. 2016;465(367-373. DOI:10.1016/j.aquaculture.2016.09.032

[2] Reverter M., Sarter S., Caruso D., et al. Aquaculture at the crossroads of global warming and antimicrobial resistance. Nat Commun. 2020;11(1):1870. DOI:10.1038/s41467-020-15735-6

[3] Dong H.T., Techatanakitarnan C., Jindakittikul P., et al. *Aeromonas jandaei* and *Aeromonas veronii* caused disease and mortality in Nile tilapia, Oreochromis niloticus (L.). J Fish Dis. 2017;40(10):1395-1403. DOI:10.1111/jfd.12617

[4] Beaz-Hidalgo R., Martinez-Murcia A., Figueras M.J. Reclassification of *Aeromonas hydrophila* subsp. *dhakensis* Huys et al. 2002 and *Aeromonas aquariorum* Martinez-Murcia et al. 2008 as *Aeromonas dhakensis* sp. nov. comb nov. and emendation of the species *Aeromonas hydrophila*. Syst Appl Microbiol. 2013;36(3):171-176. DOI:10.1016/j.syapm.2012.12.007

[5] Chen P.L., Lamy B., Ko W.C. *Aeromonas dhakensis*, an increasingly recognized human pathogen. Front Microbiol. 2016;7(793. DOI:10.3389/fmicb.2016.00793

[6] Legario F.S., Choresca C.J., Grace K., et al. Identification and characterization of motile *Aeromonas* spp. isolated from farmed Nile tilapia (*Oreochromis niloticus*) in the Philippines. J Appl Microbiol. 2023;134(12):DOI:10.1093/jambio/lxad279

[7] Xu X., Fu H., Ouyang M., et al. Description of a pathogenic strain of *Aeromonas dhakensis* isolated from *Ancherythroculter nigrocauda* in an inland region of China. Lett Appl Microbiol. 2024;77(7):DOI:10.1093/lambio/ovae046

[8] Tomas J.M. The main Aeromonas pathogenic factors. Isrn Microbiol. 2012;2012(256261. DOI:10.5402/2012/256261

[9] Liang X., Pei T.T., Wang Z.H., et al. Characterization of lysozyme-like effector TseP reveals the dependence of type VI secretion system (T6SS) secretion on effectors in *Aeromonas dhakensis* strain SSU. Appl Environ Microb. 2021;87(12):e0043521. DOI:10.1128/AEM.00435-21

[10] Talagrand-Reboul E., Jumas-Bilak E., Lamy B. The social life of *Aeromonas* through biofilm and quorum sensing systems. Front Microbiol. 2017;8(37. DOI:10.3389/fmicb.2017.00037

[11] Patil H.J., Benet-Perelberg A., Naor A., et al. Evidence of increased antibiotic resistance in rhylogenetically-diverse *Aeromonas* isolates from semi-intensive fish ponds treated with antibiotics. Front Microbiol. 2016;7(1875. DOI:10.3389/fmicb.2016.01875

[12] Santos L., Ramos F. Antimicrobial resistance in aquaculture: current knowledge and alternatives to tackle the problem. Int J Antimicrob Ag. 2018;52(2):135-143. DOI:10.1016/j.ijantimicag.2018.03.010

[13] Deng Y., Wu Y., Jiang L., et al. Multi-drug resistance mediated by class 1 integrons in *Aeromonas* isolated from farmed freshwater animals. Front Microbiol. 2016;7(935. DOI:10.3389/fmicb.2016.00935

[14] Sherif A.H., Kassab A.S. Multidrug-resistant *Aeromonas* bacteria prevalence in *Nile tilapia* broodstock. Bmc Microbiol. 2023;23(1):80. DOI:10.1186/s12866-023-02827-8

[15] Fernandez-Bravo A., Figueras M.J. An update on the genus *Aeromonas*: taxonomy, epidemiology, and pathogenicity. Microorganisms. 2020;8(1):DOI:10.3390/microorganisms8010129

[16] Lefort V., Desper R., Gascuel O. FastME 2.0: A comprehensive, accurate, and fast distance-based phylogeny inference program. Mol Biol Evol. 2015;32(10):2798-2800. DOI:10.1093/molbev/msv150

[17] Wragg P., Randall L., Whatmore A.M. Comparison of Biolog GEN III MicroStation semi-automated bacterial identification system with matrix-assisted laser desorption ionization-time of flight mass spectrometry and 16S ribosomal RNA gene sequencing for the identification of bacteria of veterinary interest. J Microbiol Meth. 2014;105(16-21. DOI:10.1016/j.mimet.2014.07.003

[18] Yin D., Guo Y., Han R., et al. A modified Kirby-Bauer disc diffusion (mKB) method for accurately testing tigecycline susceptibility: a nation-wide multicenter comparative study. J Med Microbiol. 2023;72(8):DOI:10.1099/jmm.0.001671

[19] Kolmogorov M., Yuan J., Lin Y., et al. Assembly of long, error-prone reads using repeat graphs. Nat Biotechnol. 2019;37(5):540-546. DOI:10.1038/s41587-019-0072-8

[20] Wick R.R., Judd L.M., Gorrie C.L., et al. Unicycler: Resolving bacterial genome assemblies from short and long sequencing reads. Plos Comput Biol. 2017;13(6):e1005595. DOI:10.1371/journal.pcbi.1005595

[21] Walker B.J., Abeel T., Shea T., et al. Pilon: an integrated tool for comprehensive microbial variant detection and genome assembly improvement. Plos One. 2014;9(11):e112963. DOI:10.1371/journal.pone.0112963

[22] Delcher A.L., Bratke K.A., Powers E.C., et al. Identifying bacterial genes and endosymbiont DNA with Glimmer. Bioinformatics. 2007;23(6):673-679. DOI:10.1093/bioinformatics/btm009

[23] Lagesen K., Hallin P., Rodland E.A., et al. RNAmmer: consistent and rapid annotation of ribosomal RNA genes. Nucleic Acids Res. 2007;35(9):3100-3108. DOI:10.1093/nar/gkm160

[24] Chan P.P., Lin B.Y., Mak A.J., et al. tRNAscan-SE 2.0: improved detection and functional classification of transfer RNA genes. Nucleic Acids Res. 2021;49(16):9077-9096. DOI:10.1093/nar/gkab688

[25] Tatusova T., DiCuccio M., Badretdin A., et al. NCBI prokaryotic genome annotation pipeline. Nucleic Acids Res. 2016;44(14):6614-6624. DOI:10.1093/nar/gkw569

[26] Stothard P., Grant J.R., Van Domselaar G. Visualizing and comparing circular genomes using the CGView family of tools. Brief Bioinform. 2019;20(4):1576-1582. DOI:10.1093/bib/bbx081

[27] Richter M., Rossello-Mora R., Oliver G.F., et al. JSpeciesWS: a web server for prokaryotic species circumscription based on pairwise genome comparison. Bioinformatics. 2016;32(6):929-931. DOI:10.1093/bioinformatics/btv681

[28] Kanehisa M., Sato Y., Kawashima M., et al. KEGG as a reference resource for gene and protein annotation. Nucleic Acids Res. 2016;44(D1):D457-D462. DOI:10.1093/nar/gkv1070

[29] Galperin M.Y., Makarova K.S., Wolf Y.I., et al. Expanded microbial genome coverage and improved protein family annotation in the COG database. Nucleic Acids Res. 2015;43(Database issue):D261-D269. DOI:10.1093/nar/gku1223

[30] Lombard V., Golaconda R.H., Drula E., et al. The carbohydrate-active enzymes database (CAZy) in 2013. Nucleic Acids Res. 2014;42(Database issue):D490-D495. DOI:10.1093/nar/gkt1178

[31] Urban M., Cuzick A., Seager J., et al. PHI-base: the pathogen-host interactions database. Nucleic Acids Res. 2020;48(D1):D613-D620. DOI:10.1093/nar/gkz904

[32] Chen L., Zheng D., Liu B., et al. VFDB 2016: hierarchical and refined dataset for big data analysis--10 years on. Nucleic Acids Res. 2016;44(D1):D694-D697. DOI:10.1093/nar/gkv1239

[33] Alcock B.P., Huynh W., Chalil R., et al. CARD 2023: expanded curation, support for machine learning, and resistome prediction at the Comprehensive Antibiotic Resistance Database. Nucleic Acids Res. 2023;51(D1):D690-D699. DOI:10.1093/nar/gkac920

[34] Bartie K.L., Ngo T., Bekaert M., et al. *Aeromonas hydrophila* ST251 and *Aeromonas dhakensis* are major emerging pathogens of striped catfish in Vietnam. Front Microbiol. 2022;13(1067235. DOI:10.3389/fmicb.2022.1067235

[35] Truong N., Nguyen Q., Voong P.V., et al. Genomic characterization of *Aeromonas* spp. isolates from striped catfish with motile *Aeromonas* septicemia and human bloodstream infections in Vietnam. Microb Genomics. 2024;10(5):DOI:10.1099/mgen.0.001248

[36] Xu X., Fu H., Ouyang M., et al. Description of a pathogenic strain of *Aeromonas dhakensis* isolated from *Ancherythroculter nigrocauda* in an inland region of China. Lett Appl Microbiol. 2024;77(7):DOI:10.1093/lambio/ovae046

[37] Zhao C., Qin P., Li S., et al. Exploring *Aeromonas dhakensis* in Aldabra giant tortoises: a debut report and genetic characterization. Bmc Microbiol. 2024;24(1):76. DOI:10.1186/s12866-024-03203-w

[38] Soto-Rodriguez S.A., Lozano-Olvera R., Garcia-Gasca M.T., et al. Virulence of the fish pathogen *Aeromonas dhakensis*: genes involved, characterization and histopathology of experimentally infected hybrid tilapia. Dis Aquat Organ. 2018;129(2):107-116. DOI:10.3354/dao03247

[39] Truong N.H.M., Nguyen Q., Voong P.V., et al. Genomic characterization of *Aeromonas* spp. isolates from striped catfish with motile Aeromonas septicemia and human bloodstream infections in Vietnam. Microb Genomics. 2024;10(5):DOI:10.1099/mgen.0.001248

[40] Yardimci B., Aydin Y. Pathological findings of experimental *Aeromonas hydrophila* infection in Nile tilapia (*Oreochromis niloticus*). Ankara Univ Vet Fak. 2011;58(1):47-54. DOI:10.1501/Vetfak_0000002448

[41] Bartie K.L., Ngo T., Bekaert M., et al. *Aeromonas hydrophila* ST251 and *Aeromonas dhakensis* are major emerging pathogens of striped catfish in Vietnam. Front Microbiol. 2022;13(1067235. DOI:10.3389/fmicb.2022.1067235

[42] Erickson V.I., Khoi L.M., Hounmanou Y., et al. Comparative genomic analysis of *Aeromonas dhakensis* and *Aeromonas hydrophila* from diseased striped catfish fingerlings cultured in Vietnam. Front Microbiol. 2023;14(1254781. DOI:10.3389/fmicb.2023.1254781

[43] Matys J., Turska-Szewczuk A., Sroka-Bartnicka A. Role of bacterial secretion systems and effector proteins - insights into *Aeromonas* pathogenicity mechanisms. Acta Biochim Pol. 2020;67(3):283-293. DOI:10.18388/abp.2020_5410

[44] Cherrak Y., Flaugnatti N., Durand E., et al. Structure and activity of the type VI secretion system. Microbiol Spectr. 2019;7(4):DOI:10.1128/microbiolspec.PSIB-0031-2019

[45] Cianfanelli F.R., Alcoforado D.J., Guo M., et al. VgrG and PAAR Proteins define distinct versions of a functional type VI secretion system. Plos Pathog. 2016;12(6):e1005735. DOI:10.1371/journal.ppat.1005735

[46] Spinola-Amilibia M., Davo-Siguero I., Ruiz F.M., et al. The structure of VgrG1 from *Pseudomonas* *aeruginosa*, the needle tip of the bacterial type VI secretion system. Acta Crystallogr D. 2016;72(Pt 1):22-33. DOI:10.1107/S2059798315021142

[47] Da S.F.A., Marchaukoski J.N., Raittz R.T., et al. Prediction and analysis in silico of genomic islands in *Aeromonas hydrophila*. Front Microbiol. 2021;12(769380. DOI:10.3389/fmicb.2021.769380

[48] Chen H.X., Chen F.J., Zhou Q.J., et al. Two colistin resistance-producing *Aeromonas* strains, isolated from coastal waters in Zhejiang, China: characteristics, multi-drug resistance and pathogenicity. Front Microbiol. 2024;15(1401802. DOI:10.3389/fmicb.2024.1401802

[49] Cooper C., Legood S., Wheat R.L., et al. H-NS is a bacterial transposon capture protein. Nat Commun. 2024;15(1):7137. DOI:10.1038/s41467-024-51407-5

[50] Jiang X., Li H., Ma J., et al. Role of Type VI secretion system in pathogenic remodeling of host gut microbiota during *Aeromonas veronii* infection. Isme J. 2024;18(1):DOI:10.1093/ismejo/wrae053

[51] Bartie K.L., Desbois A.P. *Aeromonas dhakensis*: A Zoonotic Bacterium of Increasing Importance in Aquaculture. Pathogens. 2024;13(6):DOI:10.3390/pathogens13060465

**Figure Legends**

**Figure 1.** Comprehensive characterization of *Aeromonas dhakensis* isolated from diseased channel catfish. (A) Gross pathology showing extensive skin lesions and fin congestion, with inset displaying maxillary ulceration (red box). Scale bar: 1 cm. (B) Tree inferred from GBDP distances calculated from 16S rDNA gene sequences. The branch lengths are scaled in terms of GBDP distance formula d5. The numbers above branches are GBDP pseudo-bootstrap support values > 60% from 100 replications, with an average branch support of 79.1%. The tree was rooted at the midpoint. Scale bar represents 0.01 substitutions per nucleotide position. (C) Metabolic profile of *A. dhakensis* CWH5 based on Biolog GEN III MicroPlates™. Heatmap displays substrate utilization patterns with purple indicating positive response, blue showing weak response, and gray denoting negative response. (D) Kaplan-Meier survival curves of channel catfish over 10 days post-infection following intraperitoneal injection with *A. dhakensis* at concentrations of 1×10^7^, 1×10^6^, 1×10^5^, 1×10^4^ CFU/fish, and PBS control.

**Figure 2.** Gross pathology and histopathological examination of channel catfish infected with *A. dhakensis* CWH5. (A) External presentation showing severe skin lesions, scale shedding, and fin congestion. Scale bar: 1 cm. (B) Abdominal cavity revealing severe intestinal inflammation, congestion, and mild intussusception (arrow). (C) Liver section from healthy control catfish displaying normal hepatic architecture with organized hepatocyte cords, clear sinusoidal spaces, and uniform nuclear morphology. (D) Liver section from infected fish exhibiting extensive hepatocellular necrosis (thin arrow) with pyknotic nuclei and cell swelling, disrupted cord arrangement, sinusoidal congestion (yellow arrows), and inflammatory cell infiltration (thick arrow). (E) Control gill section showing normal architecture with distinct primary filaments and secondary lamellae, regular epithelial cells, and patent blood spaces. (F) Gill section from infected fish demonstrating severe structural alterations including secondary lamellar hyperplasia and fusion (thin arrow), epithelial proliferation (yellow arrows), and marked filament clubbing with inflammatory response (thick arrow). (G) Control fin ray section displaying organized epithelial layers, intact connective tissue, and normal vasculature. (H) Longitudinal fin ray section from infected fish showing extensive tissue erosion, inflammatory cell infiltration (yellow arrow), structural disruption with tissue loss and vacuolation (thick arrows), and connective tissue matrix degradation (thin arrow). H&E stain; scale bar: 200 μm.

**Figure 3.** Genomic analysis and phylogenetic of *A. dhakensis* strain CWH5. (A) Circular genome map of *A. dhakensis* CWH5. From outermost to innermost: open reading frames (ORFs) on forward strand (light green), ORFs on reverse strand (dark green), GC content (black), GC skew (purple/yellow). Inner circles represent RNA genes (rRNA, tRNA, ncRNA) and mobile genetic elements (CRISPR, prophage regions). The scale indicates genome position in Mb. (B) Average Nucleotide Identity (ANI) heatmap comparing *A. dhakensis* CWH5 with closely related *Aeromonas* species. Color intensity corresponds to ANI values, with red indicating higher similarity. Scale ranges from 85% to 100% identity. (C) Tree inferred from GBDP distances calculated from genome sequences. The branch lengths are scaled in terms of GBDP distance formula d5. The numbers above branches are GBDP pseudo-bootstrap support values > 60% from 100 replications, with an average branch support of 95.6%. The tree was rooted at the midpoint. Scale bar represents 0.010 substitutions per nucleotide position.

**Figure 4.** Functional genomics analysis of *A. dhakensis* CWH5. (A) Gene Ontology (GO) classification of predicted genes. Bars represent the number of genes assigned to each GO category, divided into three main domains: biological process (blue), cellular component (red), and molecular function (green). (B) KEGG pathway classification of predicted genes. The *x*-axis shows the number of genes, while the *y*-axis lists the KEGG pathways. Colors represent different functional categories. (C) Carbohydrate-Active enZymes (CAZy) classification of predicted genes. The *x*-axis shows CAZy families, and the *y*-axis represents the number of genes in each family. GT: Glycosyl Transferases; PL: Polysaccharide Lyases; CE: Carbohydrate Esterases; AA: Auxiliary Activities; CBM: Carbohydrate-Binding Modules; GH: Glycoside Hydrolases. (D) Clusters of Orthologous Groups (COG) functional classification of predicted genes. The *x*-axis represents COG categories, and the *y*-axis shows the percentage of genes in each category. Colors correspond to different functional classes. (E) Pathogen-Host Interaction (PHI) database analysis. The *x*-axis represents different PHI categories, and the *y*-axis shows the number of genes in each category. Colors correspond to different functional classes related to pathogen-host interactions.

**Figure 5.** Genomic characterization of antibiotic resistance and virulence determinants in *A. dhakensis* CWH5. (A) Distribution of antibiotic resistance genes across different drug classes. The x-axis represents various antibiotic categories, while the y-axis shows the number of genes associated with each class. (B) Radar chart showing the relative abundance of antibiotic resistance mechanisms in *A. dhakensis* CWH5. The chart highlights the dominance of antibiotic efflux mechanisms. (C) Distribution of virulence factors according to the Virulence Factor Database (VFDB) categories. The *x*-axis displays VFDB categories, and the *y*-axis shows the number of genes in each category. (D) Subclassification of identified virulence factors. The *x*-axis shows subclasses of virulence factors, and the *y*-axis represents the number of genes in each subclass. (E) Predicted genomic islands in the CWH5 draft genome, with integrated predictions from multiple methods shown in red circles (IslandPath-DIMOB, SIGI-HMM, and IslandPick), IslandPath-DIMOB predictions in orange circles, and SIGI-HMM predictions in dark blue circles. (F) Genetic organization of antibiotic resistance genes and virulence factors within genomic islands. Arrows indicate gene orientation and function: red (virulence/resistance), blue (regulation), yellow (secretion), and gray (hypothetical/other), with corresponding gene names below.
